# Creating, publishing, and spreading processes of health-related contents in internet news sites: evaluation of the opinions of actors in health communication

**DOI:** 10.3389/fpubh.2024.1370343

**Published:** 2024-07-30

**Authors:** Eray Öntaş, Şevkat Bahar-Özvarış, Burcu Şimşek

**Affiliations:** ^1^Department of Public Health, Ankara University Faculty of Medicine, Ankara, Türkiye; ^2^Department of Public Health, Hacettepe University Faculty of Medicine, Ankara, Türkiye; ^3^Department of Communication Sciences, Hacettepe University Faculty of Communication, Ankara, Türkiye

**Keywords:** public health, health communication, online news, misinformation, science communication, infodemic, infodemiology, internet

## Abstract

**Introduction:**

The accuracy and reliability of health information disseminated through news is crucial, as it directly impacts both individual and societal health outcomes. This study aims to analyze the publication process of health content in Türkiye and its implications for public health. By examining the perspectives of various health communication stakeholders, the study seeks to identify existing issues and propose potential solutions.

**Methods:**

The research uses a mixed-methods approach, including baseline content analysis of 846 news by 133 criteria, quantitative research with 78 participants encompassing bureaucrats, academics, journalists, and health association members, and 15 in-depth interviews for comprehensive insights.

**Results:**

The content analysis indicated that 23.2% of the analyzed news articles lacked credible sources, while 63% did not mention the author’s name. A striking 96.2% of respondents stated that inaccurate health news poses a risk to public health, emphasizing the urgent need for standardized reporting practices. The majority (90.9%) pinpointed the media as the primary catalysts for infodemic spread, with 93.5% citing gatekeepers as barriers to accurate information. Eroding trust in media, fueled by unethical practices, harms both media credibility and effective public health interventions.

**Discussion:**

The study underscores the necessity for a collaborative approach among public institutions, academia, and media, focusing on responsibility, regulation, and sanctions against the infodemic. The research advocates for a balanced approach that prioritizes health rights and press freedom within a stakeholder-driven framework, highlighting that legislation alone cannot fully enhance the digital information ecosystem.

## 1 Introduction

World Health Organization (WHO) states, “The extension to all peoples of the benefits of medical, psychological and related knowledge is essential to the fullest attainment of health” (1). In the digital age, providing accurate, clear, unbiased, up-to-date, and evidence-based health information to the public is critical in all aspects of health (2). The lack of access to essential health information, significantly influences morbidity and mortality rates, particularly in low to middle-income countries and among vulnerable populations worldwide (3). This condition arises when individuals, healthcare professionals, or policymakers lack the necessary health information to protect their own health or that of others, leading to what is termed “health information poverty” (4). Its detrimental effects, in turn, have negative impacts on the health of populations, which include poor levels of health education, challenges in reaching or understanding vital health information, inadequate critical information literacy skills, and an increased susceptibility to misinformation. Digital platforms’ health information is, more often than not, biased and not credible, possibly impacting public health intervention outcomes negatively (5, 6).

The use of information technology presents a paradoxical view in the context of improving health, as it is both a part of the problem and a component of the solution (7). Currently, 64.4% of the global population uses the Internet, and 59.4% are engaged in social media (8). Türkiye’s digital landscape, where 71.4 million individuals are internet users (83.4% of the population), 62.6 million (73.1% of the population) engage actively on social media, and with a staggering 95.4% of the adult demographic using smartphones represents a critical juncture for examining health communication dynamics. The average time spent on the Internet on any device is 7 h and 57 min a day, while on social media, the average is 2.57 h a day, highlighting the pivotal role of digital platforms in both active and passive health information acquisition (9).

Due to its widespread use, information technology plays an important role in the active and passive information acquisition process: Information from these sources can be actively acquired as part of health information search behaviors for purposes such as obtaining information about a medical condition, medication, testing, treatment, understanding the cause of health-related changes, symptoms, changing behavior or daily routine, getting information on a doctor or health institution, and dealing with an existing medical condition; on the other hand, information on social media and internet news sites can be passed on to individuals by chance or incidental exposure, causing them to be passively informed (3, 4, 10, 11). Just as the lack of quality information, the quantitatively large amount of health-related misinformation spread from internet sources also deepens the health information poverty (12).

Today, digital mass media are used with increasing momentum to eliminate the information gap. As delineated by the Turkish Statistical Institute in its Household Information Technology Usage Survey (2023), over the past 3 months, a significant 61.4% of internet users accessed online news, while 66.3% sought health-related information (e.g., injury, disease, nutrition, improving health, etc.) (13). These figures underscore the internet’s role as the preeminent source for news and health information in Türkiye, with an engagement rate for news access reaching 75% (14).

As delineated in the literature, the propagation of health-related misinformation on topics such as vaccines, medications, nutrition, cancer, HIV/AIDS, outbreaks pertinent to Ebola and H1N1, tobacco, and e-cigarettes, constitutes a menace to public health (6, 15, 16). During the COVID-19 pandemic, a significant crisis of trust in information has emerged. Individuals, caught in a state of “confusion” due to unclear information and uncertain sources, now approach even reputable sources with skepticism. Despite the vast availability of information, there is a noticeable decline in the acceptance of shared truths, which are crucial for societal decisions. This has led to the fragmentation of society into “truth publics,” where parallel realities and narratives proliferate within echo chambers. Consequently, the burden of truth establishment has been shifted to organizations characterized by weak transparency and accountability bases. This unethical accountability tendency may in the end breed a long-lasting disinterest or apathy that will make it easy to experience alienation from society’s norms and values (17, p. 10). Other research has shown that, compared to correct health information, this misinformation is more likely to spread and diffuse in online contexts, adding the urgency of countermeasures and difficulty in controlling it (18). The “dilemma of trust” around science, using media as the primary channel to reach the public, could significantly endanger the diffusion of correct health information based on evidence.

While information and communication technologies (ICT) represent essential ingredients of our modern societies and economies, at the same time, they have the potential to deepen digital inequalities. The fact that ICTs can be used to exclude particular populations from services based on new technologies, such as e-government, ICT-based health, or education, is actual indeed. Socioeconomic inequalities thus influence the type and quality of practical and scientific knowledge acquisition by different groups, particularly in the context of public health issues (19). For instance, it is shown by communication theories, including the “knowledge gap” hypothesis, that disparities in information access can mirror those in wealth, leading to unequal distributions of knowledge within society. According to this hypothesis, people who continuously access information through mass media or the internet are often better informed than those not accessing them, increasing their level of knowledge regarding social contrasts of expertise (20). During the development of digital technologies, this difference has not only remained between them but also increased (21). “Digital divide” is often segmented into three clearly defined levels in research of this phenomenon: access to technology, use of this access, and information literacy. Each of these levels directly influences the outcomes and effects of internet usage (22, 23). Future studies were also challenged to conduct further in-depth research into the impacts and effects of internet usage, especially in the domain of the health-related digital divide (22). Further, for this to occur, the overall social resources need to be determined to ensure the equitable provision of access to information technology and its contents by all persons and to foster the development of crucial information literacy skills (24).

The need for reliable and accurate health communication is more important than ever, given the urgent issues brought to light by the spread of infodemic and the crisis of trust. The digital divide and associated disparities in access to information exacerbate these challenges, demanding a focused response from both researchers and policymakers. Within this contextual framework, the study is structured with three primary objectives: First, to elucidate the prevailing scenario through content analysis, this initial section evaluates the health-related content featured on designated internet news sites. Second, through quantitative research, this part aims to gauge the perspectives of chosen stakeholders from diverse sectors. It assesses their sociodemographic attributes, competencies in health communication, and views on the reliability and impact of health-related content, standard publishing criteria, resource, and medium control to mitigate infodemic, oversight, and sanctions, as well as their opinions on content creation, publication, and dissemination processes. Third, the study concludes with a qualitative analysis in its final section, providing a detailed exploration of the significance of health-related content on internet news sites regarding public health. This section delves into the challenges surrounding the accuracy, reliability, and legitimacy of information, integrating insights from the previous sections to propose solutions.

## 2 Methods

### 2.1 Type of research

This research, encompassing three sections, is a descriptive investigation employing a mixed-methods approach, integrating both quantitative and qualitative research methodologies. In the first section, content analysis is conducted on internet news sites to delineate the current scenario. In the second section, quantitative research techniques are utilized, and the views of stakeholders from diverse sectors are captured via an online data collection form. Following the insights garnered from the first and second sections, in-depth interviews with stakeholders from varied sectors have been carried out in the third section.

### 2.2 Setting

In the first section dedicated to content analysis, a scrutiny of health-related content has been carried out on the following internet news sites: Sözcü - sozcu.com.tr, Hürriyet - hurriyet.com.tr, Sabah - sabah.com.tr, Milliyet - milliyet.com.tr, Habertürk - haberturk.com; Voice of America Turkish (VOA TR) - amerikaninsesi.com, BBC News Turkish (BBC TR) - bbc.com/turkce, Sputnik Turkey (Sputnik TR) - tr.sputniknews.com, Deutsche Welle Turkish (DW TR) - dw.com/tr, Bianet - bianet.org, NTV - ntv.com.tr. In the subsequent sections, namely the Quantitative Research (2nd Section) and Qualitative Research (3rd Section), interviews have been administered both in-person and online, aligning with the COVID-19 pandemic precautions.

### 2.3 Quantitative and qualitative research sample

The section on content analysis was executed on 11 internet news sites identified above, selected through purposive sampling. These news sites were chosen based on their rankings provided on SimilarWeb’s website, a proprietary firm inaugurated in 2007 offering internet analytics services to enterprises based on composite indices like visit frequency and duration spent on the site, showcasing the most popular sites in the news/media category in Türkiye as of May 2019. The sites sozcu.com.tr, hurriyet.com.tr, sabah.com.tr, milliyet.com.tr, and haberturk.com were designated as “mainstream” media. For alternative media, news outlets financed by the United States, the United Kingdom, Russia, Germany, and Sweden, delivering news in Turkish, namely amerikaninsesi.com, bbc.com/turkce, tr.sputniknews.com, dw.com/tr, and bianet.org were chosen. Lastly, as a good practice exemplar, ntv.com.tr was selected as an online news portal whose editor has garnered accolades from professional bodies and academic entities in the realm of health communication. These internet news sites were scrutinized over a 7-day span from 16.03.2020 to 22.03.2020, with all health-related shares in text and photo gallery format containing information, recommendations, and other relevant content published throughout each day being encompassed in the analysis.

The sample for the Quantitative Research section was purposively determined, encompassing five distinct stakeholder groups engaged in health communication: bureaucrats allocated in health communication-related units within the Republic of Türkiye’s Ministry of Health (*n* = 5), two representatives each as endorsed by the Executive Boards of Professional Unions in the health sector, namely the Turkish Medical Association, Turkish Dental Association, Turkish Veterinary Medical Association, and Turkish Pharmacists Association (*n* = 8), journalists functioning as health editors or reporters in Internet News Media (*n* = 22), representatives from Medical Specialty Associations within the Coordination Board of Specialty Associations of the Turkish Medical Association (*n* = 93), and academicians who have served as advisors for theses concerning health misinformation over the last decade (2010–2020), as per the database of the Higher Education Council Presidency National Thesis Center (*n* = 27). From the envisaged total of 155 health communication actors, engagement was established with 84; amongst these actors, 78 have partaken in the research.

In the section of qualitative research, in-depth discussions were orchestrated with three individuals from each identified group, chosen predicated on their topical background and the responses they rendered to the quantitative inquiries, culminating in a total of 15 participants. Vasileiou, Barnett, et al., in their systematic analysis spanning 15 years, conducted in 2018 (18), scrutinized prevailing factors that dictate the sufficient sample size in qualitative explorations; it was discerned that saturation and pragmatic considerations were the most recurrently cited legitimacy rationales. Despite the pragmatic selection of three individuals from disparate groups within the delineated universe, saturation was perceived to have been attained nearing the culmination of the 15-participant discussions, attributed to the repetition of statements.

### 2.4 Data collection instruments and research procedure

In the Content Analysis section of our study, we implemented a comprehensive content assessment schema comprising 33 meticulously devised queries tailored to reflect both the literature and the research objectives. The schema included: 2 queries for recording the URL and headline of each news item; 13 queries for evaluating structural attributes (metadata); 7 queries for thematic analysis; and 11 queries for a holistic review of the internet news portals examined. Thematic evaluation was guided by Schema.org’s health and medical types model, which provides a structured framework for categorizing medical entities (25). Accordingly, content was thematically grouped and analyzed in relation to the Sustainable Development Goals’ health-related targets (*n* = 29) and classified based on the Global Burden of Disease Study-2017 (GBD-2017) cause hierarchy and risk, impairment, etiology, and injury n-code (REI) hierarchy (26). The news platforms were then assessed using criteria developed based on Health on the Net (HON) Codes (27). Due to the lack of standardized criteria for classifying health-related content in the existing literature, we employed a variety of specific classification criteria. This approach allowed us to clearly identify the associations within the content, using a total of 133 distinct criteria to ensure a thorough and targeted analysis.

**Figure 1:**
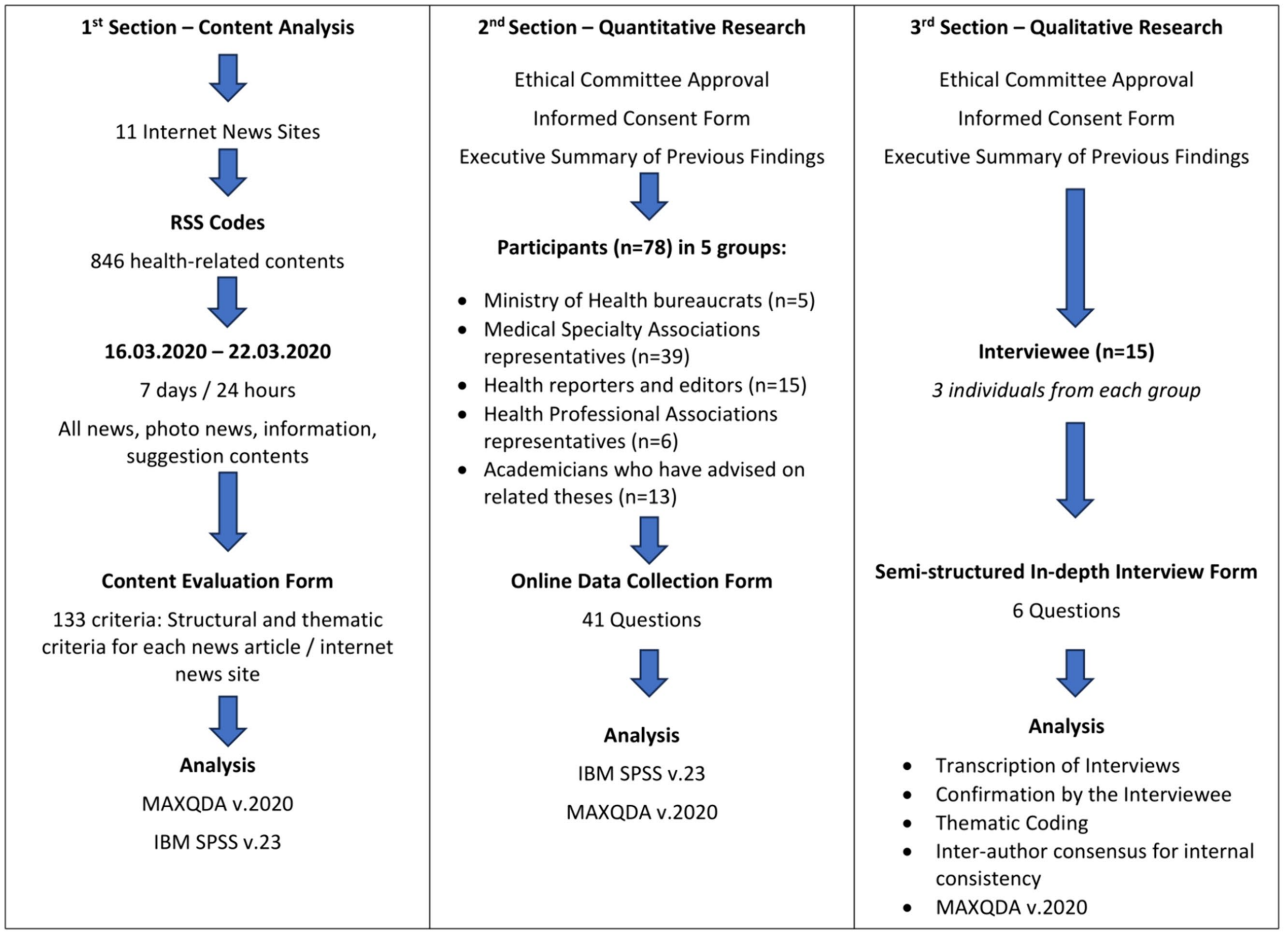
Flow diagram: overview of research methodology.

In the Quantitative Analysis section, the digital survey was designed in seven distinct segments comprising 41 questions, both multiple-choice and open-ended. These segments included: Sociodemographic Attributes (4 questions); Individual Proficiencies/Experiences in Health Communication (4 questions); Digital Media Engagement and Digital Health Literacy (13 questions); Perceptions concerning the Reliability/Legitimacy of Health-Oriented Content (4 questions); Perspectives on Health-Oriented Content within Internet News Portals (13 questions); Proposals for Resolutions (2 questions); Individual Contributions toward Resolutions (1 question). Regarding perceptions concerning the reliability and legitimacy of health-oriented content, respondents were asked to reflect on what constitutes the reliability and legitimacy of health information, characteristics that make health information accurate, what they consider to be incorrect health information, and whether they think there are verification mechanisms in place before the news is published. In terms of perspectives on health-oriented content within Internet news portals, participants were questioned on their thoughts about the risk posed by infodemic in health news, their sense of personal responsibility in combating such infodemic, and their views on whether specific standards should be maintained in health-related content on internet news sites. Additionally, the research also considered the sources that individuals believed to be the main factors of the infodemic and their opinions on whether it was necessary to have oversights and sanctions to curb the infodemic. The researchers designed the questions solely for this research purpose and were not selected from any existing scales. This approach will allow for conducting an in-depth analysis of numerous topics addressed in the seven different sections of the survey. The implementation of this approach will help capture the complex views and nuanced views about digital health communication that current scales may not be represented well. Again, with the explorative character of the survey, it aimed at collecting wide-ranging information concerning the dynamics of digital health information and not testing *a priori* hypotheses or hypotheses derived from considerations. From the insights acquired in sections 1 and 2, a semi-structured template with six key questions was applied to the Qualitative Analysis domain for open-ended discussions. These six key questions captured participants’ perception of the current status of health-related information available on news internet sites, as well as the basis of its reliability and accuracy while illuminating potential solutions and their contributions. The flowchart of the research is summarized in Figure 1.

### 2.5 Data analysis framework

The content analysis was conducted using MAXQDA v.2020 for qualitative data analysis and SPSS v.23 for quantitative data analysis. The refined results are presented in descriptive tables, showing numerical and percentage divisions. The chi-square test facilitated the comparative analysis of mainstream versus alternative media content. The quantitative analysis was executed with IBM SPSS v.23, with findings represented as numerical and percentage distributions in descriptive tabulations. When scrutinizing the correlation between descriptive and described variables, continuous variables’ distributions were probed through normality tests; the Mann Whitney-U test was employed amid determined categorical variables and those deviating from a normal distribution. Chi-square and Fisher’s Exact tests underpinned the analyses among categorical variables, with a *p*-value <0.05 deemed statistically significant. Qualitative data inquiry was conducted through MAXQDA v.2020. To bolster the rigor of qualitative analyses, a preliminary pilot study was undertaken, and the findings accrued by the observer were vetted by other investigators, selecting a 5% sample for audit.

## 3 Results

In the preliminary section where content analysis was undertaken, 11 online news outlets were assessed against 133 criteria, unearthing that amongst 846 health-related pieces: the author/responsible party was undisclosed in 63%; in 24.5% solely news agency data was divulged. The transparency concerning author/agency/responsible entity is markedly lesser in mainstream media channels (*p* < 0.05). It was discerned that 23.2% of the contents lacked source attribution. In 43.7%, a minimum of one expert viewpoint was incorporated, affirming subject-matter competence via disclosed education and specialization details; in 22.7% at least one medical practitioner’s opinion, and in 16.4% a scholarly article/report/book was cited as a source. Advisories to the readers were rendered in 71.4%. Merely in 3.5% were their open citations with web links, allowing universal access and appraisal concerning the disseminated information or data. Clickbait terminologies (cure, definitive solution, remedy, etc.) were employed in 4.4% of the headings. In thematic scrutiny, with respect to Sustainable Development Goals’ 29 targets related to health, 65.5% are related with Communicable Diseases (SDG Target 3.3). Per the GBD-2017 cause hierarchy, 63.3% are Non-communicable diseases; (COVID-19 is not encompassed in this categorization) 13.6% pertain to communicable, maternal, neonatal, and nutritional diseases. As per the GBD-2017 REI hierarchy, 33.6% are tied to environmental/occupational risks, 15.7% to behavioral risks, and 8% to metabolic risks. In 31.2%, promotion of products and/or services was observed in one or more clusters (Clusters: pharmaceutical, therapeutic, or medical merchandize; botanical product, nutritional aid; examination, surgical procedure, investigation, or protocol). While nearly all promotional contents mentioned objectives and advantages, alternatives were discussed in 49.6%, risks and side effects in 31.1%, and the advisement of “seeking physician consultation prior to utilization” was merely articulated in 14.5% (Table 1).

**Table 1:** Key findings from comprehensive analysis of health-related content in 11 online news outlets (Türkiye, 2020).

Structural criteria	*n*	%
**Authorship**		
Unknown author/responsible party	533	63.0
Known author/responsible party	313	37.0
Content creator disclosure*		
News agency name disclosed	207	24.5
Author name disclosed/no competence declared	108	12.8
Health journalist	13	1.5
Expert with declared competence	9	1.1
Source attribution for content*		
Expert opinion with declared competence	370	43.7
Public institution/official statement	211	24.9
No source attribution	196	23.2
Scholarly articles/reports/books	139	16.4
Opinion without declared competence	70	8.3
Civil society official statement	51	6.0
Private sector official statement	33	3.9
Health-related Professional organizations’ official statement	16	1.9
Other websites	15	1.8

Thematic criteria	*n*	%
Health topics classification*		
Disease or conditions	748	88.4
COVID-19	645	76.2
Risk factors	642	75.9
Prevention	636	75.2
Treatment/Therapies (including drugs and procedures)	257	30.4
Signs and symptoms	232	27.4
Studies and trials	181	21.4
Diets	138	16.3
Supplements	101	11.9
Causes	91	10.8
Tests	90	10.6
Health infrastructure	56	6.6
Exercise plans	52	6.1
Devices	46	5.4
Anatomy	31	3.7
Guidelines	6	0.7
Self diagnostic tools	5	0.6
Legal issues	3	0.4
**Promotion hidden within health-related content**		
Promoted product/service group*	264	31.2
Drug, treatment or medical product	138	16.3
Herbal product, nutritional supplement, etc.	104	12.3
Test, operation, research or procedure	27	3.2
Scope of promotional content* (*n* = 264)		
Benefits	261	98.9
Intended purpose	260	98.5
Discussion of alternatives	131	49.6
Risks and side effects	82	31.1
Advice to physician consultation prior to utilization	38	14.5

*Multiple categories can be selected for each content.

In the segment encompassing quantitative analysis, the perspectives of 78 respondents hailing from five diverse sectors were appraised, with a staggering 96.2% concurring that the current proliferation of inaccurate health information within digital news platforms poses a palpable threat to public health. The predominant catalysts for this infodemic were identified as influential personalities within the media sphere (78.2%), news agencies (60.3%), groups harboring skepticism toward health services (53.8%), and health journalists and editors (51.3%). Participants pinpointed “Media” (90.9%), content generators (76.6%), internet users (66.2%), and the deficit of coherent and accurate health information disseminated by governmental entities (49.4%) as the fundamental drivers behind the online dissemination of erroneous health insights. A significant 93.5% acknowledged an interruption in the accurate health information generation and dissemination continuum; within this disruption, 67.5% underscored the predilection of “gatekeepers/decision-makers for speculative content driven by economic and political motives over factual information,” while 53.2% accentuated the “inadequacy of adept individuals in generating accurate and publicly comprehensible information.” The realms most plagued by the distribution of incorrect health data, as perceived by 91% of respondents, are “commercial internet platforms,” followed by television productions (60.3%), and print media (51.3%). Education emerged as a paramount instrument in combatting infodemic, as endorsed by 55.1%, with 17.9% advocating for systemic alterations entailing deterrent sanctions by both public and private sectors to curb misinformation. The lack or insufficiency of verification mechanisms within publishing entities was acknowledged by 92.2%. A robust 93.6% championed the imperative of oversight to mitigate incorrect health information dissemination: the Ministry of Health (69.2%), the Turkish Medical Association (51.3%), and subject-specific Medical Specialty Associations (42.3%) were mooted as suitable overseers. The call for sanctions resonated with 92.2%, wherein 77.9% pinpointed the infodemic source, 72.7% the publishers, and 48.1% the sharers as liable entities. Upon a deeper analysis bifurcating media personnel from other stakeholders, a mere 20% of media professionals, contrasting with 54% of other actors, endorsed sanctions for misinformation purveyors, delineating a statistically substantial discrepancy (*p* = 0.022) (Table 2).

**Table 2:** Key findings from qualitative analysis of opinions on actors in health communication (*n* = 78).

Theme	Perception	Description	%
Impact of infodemic on public health	High risk to public health	Belief that inaccurate health information in news poses a risk to public health	96.2
	Risk to public health in certain conditions	Belief that inaccurate health information in news poses a risk to public health, in certain conditions	3.8
Sources of infodemic*	Media influencers	High-profile individuals in the media	78.2
	News agencies		60.3
	Healthcare skeptic groups		53.8
	Health reporters/editors		51.3
	Health professionals		17.9
	Public officials		14.1
	Civil society organizations		5.1
Catalysts for infodemic spread*	Media	Selective impact by media gatekeepers, economic concerns in supply due to demand	90.9
	Content creators	Inadequacy in producing public beneficial information by competent individuals and organizations, unmet demand	76.6
	Internet users	Need for health information search behavior, lack of critical skills due to unmet information gaps	66.2
	Public institutions	Insufficient accurate and understandable health information provided	49.4
	Healthcare services	Inadequate communication duration between service provider and receiver	41.6
	Social media companies		1.3
Barriers to accurate information*	Gatekeepers/decision-makers	Preference for speculative content over accurate information for economic and political reasons	67.5
	Competent individuals	Not producing enough correct and understandable information for the public	53.2
	Information not reaching gatekeepers/decision-makers		32.5
	Demand not met by users even if correct information is produced and published		32.5
	Inability to discern right from wrong		31.2
Media for infodemic spread*	Commercial internet platforms		91.0
	Television productions		60.3
	Press/Newspapers		51.3
	Internet forums		48.7
	Instant messaging applications		47.4
Countering health infodemic	Education	Emphasizing the need for health literacy to discern misinformation	55.1
	System change for deterrence	Need for deterrent sanctions by the private sector and public to prevent misinformation	17.9
	Verification mechanisms	Detecting and correcting misinformation	15.4
	System change for regulation	Need for regulatory actions by the private sector and public to prevent misinformation	7.7
	Tools	Helping users to discern misinformation	2.6
Oversight*	Ministry of health		69.2
	Turkish medical association		51.3
	Medical specialist associations		42.3
	Information technologies and communications authority		26.9
	An independent organization		24.4
	Radio and television supreme council		21.8
	Press/Journalists		15.4
	Civil society organizations		7.7
	The user themselves		6.4
	Commercial internet platforms		3.8
	Consumer arbitration boards		2.6
Sanctions for infodemic*	Against misinformation source		77.9
	Against publisher of misinformation		72.7
	Against sharer of misinformation		48.1
	No sanctions needed		7.8

*Multiple responses can be selected for this question.

All participants exhibited consensus on the necessity of adhering to certain standards while generating health-related content on internet news platforms. The percentage of agreement concerning the delineated standards is documented in the Table 3.

**Table 3:** Proposed standards for health-related content on internet news sites.

Criteria	Frequency (*n*)	Percentage (%)
**Author name and relevant expertise** *(n = 78)*	78	**100.0**
**Recency** *(Date of information acquisition, last updated date) (n = 78)*	78	**100.0**
**Citation and verifiability** *Accessible references to data sources, provision of balanced evidence addressing different aspects of the topic (n = 78)*	77	98.7
**Completeness statement** *Declaration that the health information provided is to support, not replace, doctor-patient relationships, and consultation with a physician is necessary for the appropriateness of the information (n = 78)*	75	96.2
**Readability** *Simple and understandable expressions; explanatory infographics and tables (n = 77)*	73	94.8
**Privacy statement** *Transparency about the usage and security procedures of user-collected data (n = 76)*	71	93.4
**Ethical declaration** *Declaration of no vested interest by the author regarding the content (n = 77)*	71	92.2
**Contact addresses and feedback mechanism** *(n = 78)*	71	91.0
**Guidance** *Detailed resources or contact information for visitors seeking further support and current information regarding the content (n = 77)*	67	87.0
**Responsibility statement** *Declaration of author’s responsibility for any adverse situations arising from the content (n = 77)*	65	84.4
**Legal guidance** *Basic guiding information for those wishing to pursue legal rights concerning publishing and current applicable laws (n = 77)*	62	80.5

In the third section wherein, the qualitative research was undertaken, through comprehensive discussions with 15 participants across five distinct groups, it was articulated that there necessitates a “collective responsibility, apportioned among readers, media, public authorities, and the academia.” Within the media spectrum, the onus of responsibility is envisaged to reside within the “editorial chain.” The paramount responsibility is underscored to vest with the “Public Authority” to orchestrate the process on society’s behalf and to ensure the fulfillment of obligations by all societal individuals and establishments. It was highlighted that, given its direct bearing on health, media institutions should harbor a control mechanism imbued with a sense of responsibility. Apprehensions were aired regarding potential encroachments on press freedom in the presence of an external control mechanism, propelling the recommendation for the cultivation of an internal control mechanism. Pertaining to the extant scenario, foundational expectations from academia, media, public establishments, and legislators encompass a holistic approach at every juncture, meticulously delineated boundaries of health rights and press freedom, and engagement with all identified responsible stakeholders in all ensuing steps.

## 4 Discussion

A significant 96.2% of participants are of the view that the inaccurate health-related content present in today’s internet news poses a public health risk; a minority of 3 participants (3.8%) acknowledge this assertion to be true in certain scenarios. The quality of health information available online has been substantially impacted by the transformation of the Internet into a participatory and social platform with the emergence of Web 2.0 (28, 29). Wardle and Derakhshan’s paper offers a framework for analyzing information disorder, classifying it into three types: misinformation, disinformation, and malinformation, based on the accuracy of the information and the intent to harm (30). In the digital era, which is also defined by the “weaponization of mistrust” and “computational propaganda” (31), information disorder has become a serious public health concern due to the rapid increase in the speed, scale, and scope of information flow. The widespread use of the internet, social media, and mobile phones has fundamentally disrupted established business models in the news sector. New business models often grapple with budget constraints, infrastructure challenges, and a scarcity of resources, leading to a reduction in “on-the-ground,” real-life news coverage (32). The pressure to continuously create content to feed the homepage and social media accounts, along with the speed of publication demands, has reduced the quality control processes such as verification, diversity of data, and content enhancement. The blending of news and commercial information, along with the risk of eroding reader trust through hidden advertisements and “clickbait” headlines, has increased information disorder. In an increasingly competitive online world, content produced to attract visitors to websites rather than inform the public is promoted to increase digital advertising sales, sometimes at the cost of excellence and viability in journalism practice. The demand for “real-time” content increases the potential for errors and the merging of all types of media blur expertise in specialized areas. This pressure often translates into a “publish first, check later” approach (33). It becomes desperately important to enforce robust internal controls within media organizations to check the spread of non-factual information. Overcoming these challenges is possible only when media organizations and journalists base the centrality of transparency on their practice of ethical journalism and chase down evidence-based reporting. The implementation of rigorous verification processes to identify the prevalence of misinformation and thorough validation of data, sources, and digital images are necessary. Furthermore, additionally, it is essential that the framing of news agendas is consistent with the public’s requirements and benefits, thereby guaranteeing that the media act as a constructive force in society (31).

The digital shift, particularly the move to digital advertising dominated by giants like Google and Facebook, has not fully supported media organizations, compelling them to develop new business models. The research underscores social media corporations as pivotal conduits for the dissemination of health misinformation online, a viewpoint further enriched by Farkas and Schou’s discourse on “digital capitalism” (34). Delving into the underlying causality with a holistic lens, beyond the “political power” deliberated in ensuing sections, the nexus between advertising revenue distribution and content formulation in media entities warrants scrutiny. In Türkiye, during 2021, a staggering 99.2% of internet users utilized search engines within the preceding month (35), with a dominant majority (over 80%) opting for Google (36). Anticipations are rife for Google, the online advertising vanguard, to steer 29% of the global digital ad outlays in 2021, with Facebook trailing at 24% (37). Peering into the European landscape, notably the UK, a presumed ‘Duopoly’ held by these behemoths commandeers nearly 70% of the market share (38), while a ‘Digitalization and Competition Policy Report’ initiated by Türkiye’s Competition Authority in January 2021 could shed light on the analogous scenario locally (39). The year 2020 saw a purported investment of around 7.5 billion TL in digital media ventures in Türkiye. A dissection of the investment spread across ad modalities unveils that paid ad campaigns ensuring prime search engine rankings (37.9%), impression or click-centric ads (35.2%), and video ads (20.5%) are poised to engulf a substantial portion of the nearly 7 billion TL investment (40). Yet, post the 7.5% digital service tax amendment in March 2020, the revenue accrued from April 2020 to March 2021 stood at 1.66 billion, with the implicated sector boasting a transaction girth of 22 billion TL (41, 42). A foray by the Reuters Institute, encompassing 234 digital media chieftains across 43 nations, revealed that a hefty 66 and 61% acknowledged impression-based and native ads, respectively, as significant revenue streams (43). Internet news outlets, in a bid to bolster ad revenues, are veering toward marketing “content” crafted to fuel site traffic over *bona fide* “news,” employing SEO tactics like clickbait, content pagination, ‘click to continue reading’ prompts, and auto-refresh features (44). This paradigm of churning out “cheap” content, gauged by metrics like views, clicks, site duration, and shares, is embarking on a quality compromise journey, undermining public trust in securing timely, accurate, and comprehensible information. The 2021 Turkey Digital Media Report by the International Press Institute accentuates, through engagements with media moguls, that colossal platforms are swaying the publishing ecosystem by “propagating clickbait” (45). The prevailing revenue distribution algorithms are propelling large media houses with hefty SEO arsenals to eclipse other media entities in search engine visibility, thereby stifling the distribution share for outlets disseminating alternative viewpoints and local news narratives.

Media professionals, influenced by routine media practices, institutional goals, external pressures, and ideological influences - as outlined in the agenda-setting framework (46), which focuses on how media prioritize issues to shape public perceptions - actively engage in “marketing” health information. The communal benefits of disseminating critical public health information may be overshadowed by the prioritization of content that generates the most clicks, views, and shares. For instance, prevalent and often fatal diseases such as cardiovascular diseases, cancers, chronic respiratory disorders, diabetes, and chronic kidney diseases receive significant attention. Nevertheless, there is a significant inclination among internet news sites to prioritize sensationalist and ambiguous lifestyle advice over clear and actionable guidance on preventable risk factors, including the cessation of tobacco, the reduction of harmful alcohol consumption, the reduction of salt intake, the reduction of trans-fat and sugar-sweetened beverages, and the increase in physical activity (47). This method has the potential to diminish the effectiveness of disease prevention and management strategies and weaken the impact of critical public health messaging.

The research question onto the accountability for the accuracy and reliability of health-related information on internet news platforms introduces the notion of collective responsibility. In many cases, it is posited that responsibility is distributed among a number of different stakeholders, such as the reader, the source of the information, media entities, public authorities, and academic institutions, to varying degrees. In addition, a sizeable number of respondents emphasized that the public authorities bear the lion’s share of this responsibility. This is because of the role that they play in orchestrating the processes that are involved.

In Türkiye, examining the governance of the Internet reveals that the Ministry of Transport and Infrastructure set up through Decree-Law No. 655, is designated with powers concerning the electronic communication sector under Law No. 5809. Additionally, an Internet Development Board operates under this ministry, is mandated to foster a conducive environment for internet growth through research and assessments, and is entrusted with shaping the national internet policy. The Information and Communication Technologies Authority (ICTA), affiliated with the ministry via Law No. 2813, is tasked with executing the board’s decisions (48). The ICTA holds the regulatory reins in electronic communication, as outlined in Law No. 5651, which addresses the regulation of online publications and the combat against online crimes (49). Other pivotal legislations in the domain of Internet law include Law No. 5369 on Universal Service and Law No. 5809 on Electronic Communication (50, 51). At the time of this study, the outdated definitions and responsibilities in the Press Law for internet news sites, along with the lack of adherence to author identification in periodic publications, contribute to legislative gaps fostering information disorder (52).

This research discovered that 63% of the evaluated contents lacked author, agency, or responsible party identification, and some respondents pinpointed anonymous news as a significant misinformation catalyst. Unanimously, participants advocated for a standard requirement of disclosing the author’s name, their subject-matter expertise, and the creation and last update dates of the content. The necessity of standardly presenting an author’s name and credentials in every piece of content is partly driven by concerns around copyright issues. A study engaging news website editors revealed that they unanimously source information from “rival news outlets” and “social media” (53). The accountability of content providers is defined in Law No. 5651, and Law No. 5846 on Intellectual and Artistic Works extends this definition to digital transmissions in its additional article no.4 (54). However, the present regulation may fall short in deterrence, as it positions the “Notice-Takedown System” at the forefront, coupled with a 3-day timeframe allocated for the rights holder’s request. Moreover, the practice of amplifying individuals’ visibility—sometimes in sensitive scenarios—by featuring personal opinions from social media on news websites, brings the discussion of “usage permissions” and accurate attribution to the fore, a discourse evident not only in Türkiye but also in broader international dialogs (55).

Participants underscored two key considerations concerning the amendments needed for the current deficiencies: firstly, the necessity of accurately delineating the constitutional boundaries of press freedom, personal rights, and health rights while establishing legal frameworks for publications; secondly, ensuring that these legislative amendments are crafted in a collaborative manner, with extensive engagement from public, private, and civil society entities. Conversely, the global scenario paints a different picture, where many nations have faced criticism for infringing upon freedom of expression and press liberty, often justified by the ongoing pandemic (56). In the COVID-19 epoch, scrutinizing nations’ legal battles against the surging “disinformation” tide, amplified by the infodemic, reveals a spectrum of responses. For instance, new legislation categorizing disinformation as a criminal offence has emerged in countries like Hungary, Bolivia, South Africa, Botswana, Zimbabwe, and the Philippines. Additionally, instances of detentions have been reported in Kenya, the Philippines, Sri Lanka, and Cambodia, triggered by critiques of governmental approaches toward COVID-19 containment. Meanwhile, Serbia and India have instituted “directive” frameworks permitting only official or government-sanctioned COVID-19 information to be disseminated. Lastly, notable restrictions on COVID-19-related information dissemination have been imposed by authorities in China, Belarus, and Kuwait (57, 58).

The notion of “responsibility” in internet news media naturally leads to the need to define oversight and accountability. According to quantitative research findings, a significant 93.6% of participants believe that oversight is crucial to prevent misinformation related to health; 92.2% mention the lack of or inadequacy of a verification mechanism as an internal oversight process in broadcasting institutions. On the flip side, when it comes to external oversight mechanisms, participants suggest that the Ministry of Health of the Republic of Türkiye (69.2%), Turkish Medical Association (51.3%), and relevant medical specialty associations (42.3%) could be responsible for oversight, depending on the subject matter. There is an expectation from the academic community to establish oversight mechanisms, while public institutions are anticipated to organize oversight and regulatory activities. Qualitative research findings collectively emphasize that due to the direct impact of health news on individual and community health, it should be carried out with a particular sensitivity. Therefore, a sense of responsibility throughout all stages of the publication process is vital within media organizations, necessitating an internal oversight mechanism. A heavily stressed point regarding internal oversight is “professional ethics.” The ethical regulations and legislation concerning health professionals who could serve as sources have been defined by professional organizations: Law on the Practice of Medicine and Its Branches (Article 24) (59), Medical Deontology Regulation (Articles 8–9) (60), Guide on Shares of Physicians and Health Institutions in Electronic Media (61), Turkish Medical Association Principles on Physician and Drug Promotion (62), Guide on Publications of Dentists in All Communication Media (63), Turkish Dental Association and Chambers of Dentists Discipline Regulation (Article 8/a) (64) and the Regulation on Promotion and Information Activities in Health Services issued in 2023 (65).

A crucial component of internal oversight is the decision-makers at the pinnacle of the editorial chain. Research by Ioannidis highlights a shortfall in media coverage of significant public health issues and their modifiable risk factors, while individualized suggestions are prominently featured (47). Sezgin, critically examining health discourse in media, bases his assessments on the implications of neoliberal economy on healthcare systems (66). The investigation delves into the transformation in biotechnology, the pharmaceutical industry, health insurance, and the cosmetic industry under the banner of “for a healthier society,” alongside the medicalization of everyday life and physiological concepts like birth, death, menopause, and aging. The impact of gatekeepers on content selection is explored in a study by Yalçınkaya (2019) involving news site editors (33), where it’s found that editors’ judgments are influenced by their institution’s political stance, fears of political pressures, the publication policy, and the expectation of high click-through rates. Ayaz’s study unveils the ideological influences on gatekeepers’ decision-making processes, emphasizing the need for revisiting editorial independence (67, p. 278). Reports by Turkish Journalists Society (68), Turkish Journalists Union (69), Turkish Media and Law Studies Association (70), Freedom House (71), and European Commission (72, p. 37) have shed light on press freedom violations. In this context, legal frameworks should uphold press freedom, fostering a transparent structure to mitigate economic and political influences on editorial independence, and encouraging unionization (69) to rekindle a journalist’s primary accountability toward the public and truth.

When examined through the lenses of information disorder, responsibility, and oversight, a notable “legal disorder” that potentially infringes on various rights is observed. Consequently, the interviewees frequently expressed reservations about the yet-to-be-defined external oversight and punitive mechanisms under the current legal conditions, fearing they might encroach upon fundamental rights and freedoms. They advocated for the promotion and endorsement of “good practice examples” as corrective measures. Participants are looking to legislators to delineate boundaries concerning the focus of sanctions (information source, publishing institutions, social media and internet service providers, health information communication tools, sharers, advertisements); the limits of sanctions (safeguarding public health for the common good, not impeding personal freedoms, and not hindering scientific advancements); and the conditions under which they will be applied (non-scientific, commercially-driven publications, those without clear references, unethical ones). They underscored the necessity for formulating regulations directed at oversight and demonstrating steadfastness in implementing these regulations.

When comparing Türkiye’s response to the infodemic with global initiatives, certain similarities as well as distinctions become apparent. In recognition of the fact that misinformation is a substantial obstacle in the public health response to the pandemic, WHO has brought attention to the concept of an “infodemic” (73). It is important to note that WHO has established the WHO Information Network for Epidemics (EPI-WIN) in order to guarantee that communities receive trustworthy, timely, and easily understandable advice and information regarding public health events and outbreaks (74). A Public Health Research Agenda for Infodemics Management has been developed through the global collaboration of nations under the aegis of the World Health Organization (12, 75). Many contributions forming the process made by this agenda included Artificial Intelligence tools like WHO-EARS to guide social listening and identify information gaps (76).

As part of this strategy, there are numerous policies that the European Union has put in place to enhance accountability and transparency within digital communications. Some of these policies include the EU Code of Practice on Disinformation, the COVID-19 Disinformation Monitoring Programme, and the Digital Services Act, which is aimed at regulating online platforms to curb the spread of false information through strict monitoring and reporting mechanisms (77–79). Such measures are also part of the most recent legislation in Türkiye, even though it deviates significantly from the country’s policy. However, it seems that Turkey, unlike the EU member states, has focused more on legal infrastructures and strict regulations meant to oversee the distribution of such content that is realized as harmful or false.

While in the EU, independent bodies and NGOs contribute to the multi-stakeholder, decentralized approach to information oversight and verification—this is seen, for example, with the European Digital Media Observatory (80)—recent Türkiye legislative changes, such as Law No. 7418 (81), bringing state mechanisms directly into the picture through monitoring and controlling online content (82).

In addition, Türkiye’s regulations place a strong emphasis on the legal ramifications of infractions, including particular criminal penalties for disseminating false information. This goes beyond the administrative and civil remedies that are generally preferred in Western approaches (83). This divergence highlights a more stringent and controlled method in Türkiye, aiming to quickly stem the dissemination of disinformation, whereas the EU and countries like Canada and the UK’s strategies often emphasize long-term educational strategies and technological solutions to foster a more informed and resilient public (82).

### 4.1 Strengths and limitations

Our study is a pioneering investigation into the topic of “infodemic” before the WHO had formally defined the concept, thus laying the groundwork for future research in the critical area of accurate health information during a pandemic. It benefits from the collective insights of diverse fields, enhancing problem-solving and intervention strategies. Nevertheless, it has constraints. The data collection phase coincided with the announcement of the COVID-19 pandemic, overshadowing other health-related topics we intended to analyze. The pandemic also impeded direct access to health-related actors, which could potentially reduce the participation of health professionals. Due to the fact that we lacked the specialized expertise necessary to verify the factual accuracy or scientific validity of each health content, we relied on practical criteria to ensure the reliability of the information they contained. A purposive sampling approach was required due to resource constraints and the pandemic, which restricted the generalizability of our findings. Future research could resolve these limitations by incorporating broader actor participation and expanding the evaluative criteria for health-related content on internet sites.

## 5 Conclusion

This research brings forth the critical role of journalists in putting public health at the center of reporting. To effectively fight the infodemic and ensure the success of health interventions with the population, it is essential to regain trust in journalism as a sector that has always safeguarded the truth. Research shows that such efforts must be undertaken in collaboration with various stakeholders, including media, academic institutions, and regulators, to guide ethical standards and increase transparency. The paper suggests an integrative vision of health communication that brings forward the awareness of a public health agenda as fundamentally and increasingly interconnected with democratic processes, human rights, and social cohesiveness. In public health protection, public authorities play a crucial role in ensuring that all people have access to quality internet and accurate and dependable information. Supplementary Table S1 provides recommendations to the public authorities to assist the public authorities in fighting information disorder. Lastly, it is imperative for the state to undertake positive actions to facilitate the realization of the right to health and the enhancement of public health, thereby creating an environment where all members of the community can fulfill their responsibilities.

## Data availability statement

The datasets presented in this article are not readily available because the second part of the study – the quantitative research, and the third part – the qualitative research, might reveal the participants’ identities when the data is shared. Therefore, data can only be shared upon a reasonable request. Requests to access the datasets should be directed to eontas@ankara.edu.tr.

## Ethics statement

The studies involving humans were approved by the Non-Interventional Clinical Research Ethics Committee of Hacettepe University, Ankara, Türkiye under the decree number GO20/129 (Evaluation Date: 27.01.2020), 2020/03–08. The studies were conducted in accordance with the local legislation and institutional requirements. The participants provided their written informed consent to participate in this study.

## Author contributions

EÖ: Conceptualization, Data curation, Formal analysis, Investigation, Methodology, Project administration, Software, Validation, Writing – original draft, Writing – review & editing. ŞB-Ö: Conceptualization, Formal analysis, Methodology, Project administration, Supervision, Validation, Writing – review & editing. BŞ: Conceptualization, Formal analysis, Methodology, Project administration, Supervision, Validation, Writing – review & editing.
