# Supplementary material for: Creating, publishing, and spreading processes of health-related contents in internet news sites: evaluation of the opinions of actors in health communication
Source: Front Public Health. 2024 Jul 30;12:1370343. doi: 10.3389/fpubh.2024.1370343 (PMC11319292; doi:10.3389/fpubh.2024.1370343)
Supplement: Supplementary file 1 [file Table_1.pdf]

Supplementary Material

Creating, Publishing and Spreading Processes of Health-Related Contents in Internet News Sites: Evaluation of the Opinions of Actors in Health Communication

Eray Öntaş[1], Şevkat Bahar-Özvarış[2], Burcu Şimşek[3]

[1] Department of Public Health, Ankara University Faculty of Medicine, Ankara, Türkiye

[2] Department of Public Health, Hacettepe University Faculty of Medicine, Ankara, Türkiye

[3] Department of Communication Sciences, Hacettepe University Faculty of Communication, Ankara, Türkiye

*** Correspondence:** Eray Öntaş eontas@ankara.edu.tr

Citation: Öntaş E, Bahar-Özvarış Ş and Şimşek B (2024). Creating, publishing, and spreading processes of health-related contents in internet news sites: evaluation of the opinions of actors in health communication. Front. Public Health 12:1370343. doi: 10.3389/fpubh.2024.1370343

Supplementary Table 1. Recommendations to Public Authorities

**1) Formation of a Scientific Advisory Committee on Information Disorder is imperative.**	- To architect a comprehensive policy blueprint addressing information disorder, its ramifications, and containment strategies, the inception of an autonomous Scientific Advisory Committee is essential. This committee, embodying a confluence of perspectives from all relevant stakeholders, will serve as a linchpin for informed policy formulation and execution.
**2) Internet Legislation Requires Structuring.**	- A foundational framework encompassing freedom of expression, universal access to information, privacy rights, and active stakeholder participation needs to be established to fashion regulations that deter the criminalization of information disorder through fortified internal oversight mechanisms.<br>- Legislation tailored to online platforms should be drafted, calibrated based on their respective roles in the digital ecosystem, size, and impact, delineating clear-cut responsibilities and accountability. It's pivotal to demarcate boundaries judiciously during this process to avert the potential transmutation of online platforms into ideological apparatuses of the state.<br>- Proactive measures to curb monopolization within the digital ecosystem are imperative, coupled with the orchestration of competitive conditions.<br>- Pertaining to the distribution of advertising revenue, especially within health-centric content, it's essential to articulate criteria that gauge the caliber of news generated by internet news outlets, diverging from conventional metrics like view count, click rate, on-site duration, and sharing frequency.<br>- Activation of the Internet Development Board is crucial, with legislation framing objective criteria to ensure a well-rounded representation of all stakeholders. Moreover, the alignment of internet-related regulations with international standards and scientific evidence is a requisite for fostering a robust digital landscape.

**3) Refinement of Press Legislation is Crucial.**	- Upholding press freedom is imperative.<br>- Regulatory undertakings aimed at aligning publications with legal statutes must judiciously delineate the contours of constitutionally protected press freedom, personal rights, and health rights. The orchestration of legislative amendments should be a collaborative venture, embracing extensive involvement from public sector entities, private sector counterparts, and civil society organizations.<br>- The ambit of the Press Law and Press Work Law should be expanded to encompass internet news media and its personnel.<br>- The establishment of an autonomous oversight entity, encapsulating representation from academia, fact-verification entities, and all pertinent stakeholders is essential. This body should proactively disseminate reports and advisories concerning information disarray and rights infractions, foster interdisciplinary engagements in prioritized domains, formulate and oversee the adherence to standards aimed at elevating the quality and trustworthiness of health-centric content.<br>- Safeguarding the heterogeneity and viability of the internet news media ecosystem is vital. By bolstering non-mainstream media entities, a myriad of perspectives can be showcased, and backing local media outlets is key to averting the emergence of "news deserts."<br>- Enactment of regulations to amplify engagement within professional bodies should be pursued to nurture professional rights. Support should be extended to professional entities delving into rights-driven advocacy and capacity augmentation endeavors, coupled with the instauration of an internal oversight mechanism to curb unethical professional practices within these organizations.
**4) Augmentation of Risk Communication is Essential in Addressing Public Health Challenges and Mitigating Epidemic Outbreaks.**	- Establishment of a Health Communication Advisory Board is pivotal. By refining risk communication strategies, a bolstered level of trust between the community and public institutions can be fostered.<br>- Legislative amendments delineating the onus of public and private sector entities in championing transparency and disseminating public information are imperative. Additionally, nurturing an open data culture stands as a critical pillar in this endeavor.<br>- The orchestration of recurrent informational and feedback-driven engagements with representatives from media organizations is instrumental in ensuring a two-way flow of critical insights and updates.
**5) Proliferation of Enlightening Material on Core Health Issues is Imperative.**	- Augmentation in the capability to generate and disseminate health information that is anchored in evidence, accuracy, reliability, and contemporaneity is pivotal.<br>- Provision of "timely, accurate, and comprehensible advice and information from trustworthy sources" to meet public exigencies should be orchestrated via dedicated public information platforms.<br>- Fostering specialized collaborative endeavors that can serve as a reservoir of credible information for social media platforms and internet news outlets is essential.
**6) Embark on a Campaign for Fostering Critical Literacy.**	- A comprehensive literacy embracing health, media, information, technology, economics, and law should be compulsorily integrated into the basic education curriculum. Ensuring the enlistment of adept professionals for imparting this education is paramount, alongside an ongoing surveillance and enhancement of the educational quality.<br>- A thorough assessment should be undertaken to ascertain the requisites of demographics that missed out on this educational provision during their

	foundational learning phase. Tailored programs aimed at children, adolescents, the elderly, and other susceptible groups should be meticulously orchestrated.
**7) Foster the Promotion of Exemplary Practices.**	- Incentives should be provided to internet news sites and online platforms that are dedicated to the production, enhancement, and dissemination of high-quality news.
**8) Promote Scientific Exploration to Combat Information Disorder.**	- Establish transparent data sharing protocols by designating responsible personnel within the Health Statistics and Causal Analyses (SINA) program.<br>- Streamline the ethical approval process for COVID-19 related research by eliminating the prerequisite of obtaining approval reports from the COVID-19 Scientific Research Evaluation Commission under the General Directorate of Health Services of the Ministry of Health of the Republic of Turkey, prior to the ethical review by respective committees.
**9) Foster the Development of a Digital Surveillance System.**	- Drawing inspiration from the WHO's GOARN (Global Outbreak Alert and Response Network), it's imperative to embed an "infodemiology" framework within the National Health Threat Early Warning and Response Network to facilitate nuanced analysis of information and communication patterns.
